# Biological Responses to Local Vibratory Stimulation for the Lower Legs and Lower Back and Criterion Values Based on Sweep Frequencies of Healthy Individuals: An Observational Study

**DOI:** 10.3390/healthcare11162243

**Published:** 2023-08-09

**Authors:** Keitaro Kawai, Yoshiji Kato, Tadashi Ito, Kazunori Yamazaki, Jo Fukuhara, Yoshihito Sakai, Yoshifumi Morita

**Affiliations:** 1 Department of Electrical and Mechanical Engineering, Graduate School of Engineering, Nagoya Institute of Technology, Nagoya 466-8555, Japan; k.kawai.750@nitech.jp (K.K.); j.fukuhara.176@nitech.jp (J.F.); 2 Department of Physical Therapy, Nagoya Women’s University, Nagoya 467-8610, Japan; yoshi-waterfalls@cam.hi-ho.ne.jp; 3 Three-Dimensional Motion Analysis Room, Aichi Prefectural Mikawa Aoitori Medical and Rehabilitation Center for Developmental Disabilities, Okazaki 444-0002, Japan; sanjigen@mikawa-aoitori.jp; 4 Department of Integrated Health Sciences, Graduate School of Medicine, Nagoya University, Nagoya 461-8673, Japan; 5 Institutional Research Center, Aichi Mizuho College, Nagoya 467-0867, Japan; kn-yamazaki@mizuho-c.ac.jp; 6 Department of Orthopedic Surgery, National Center for Geriatrics and Gerontology, Obu 474-8511, Japan; jsakai@ncgg.go.jp

**Keywords:** proprioceptive function, local vibratory stimulation, sweep frequency, cutoff values

## Abstract

Declining proprioceptive function is associated with problems such as lower back pain and falls. Therefore, we developed a vibration device using sweep frequency to evaluate several proprioceptors with different response frequency ranges. This study aimed to elucidate the biological responses of healthy individuals to vibratory stimulation at different sites and frequency ranges and to propose cutoff values to determine the decline in proprioceptive function. Mechanical vibration was separately applied to the lower legs and lower back, and proprioceptive function was evaluated by defining the ratio of the center of pressure (CoP) in the anteroposterior direction during mechanical vibration to that during no vibration in the three frequency ranges. The cut-off value was defined as the mean value, with the standard deviation subtracted for each indicator. The cut-off values were higher in the lower legs than in the lower back at all frequency ranges and in the 30–53 Hz and 56–100 Hz frequency ranges for both the lower legs and lower back. In healthy individuals, 9.9% and 8.6% were below the cut-off values in the 30–53 Hz and 56–100 Hz frequency ranges for the lower legs, respectively.

## 1. Introduction

Poor proprioception may be related to a variety of problems, including lower back pain and falls [1,2,3,4,5,6,7]. Proprioceptors are deep sensory organs that detect the position of sites, the state of movement and muscle contraction, and resistance and mass applied to the body. They are important for postural control [8]. In particular, the proprioceptors of the lower legs and back aid in postural control. Various methods have been devised to evaluate these proprioceptors [9,10,11,12,13]; however, mechanical vibration application [14,15,16] and postural control measurements are generally used owing to the need for an integrated approach using postural control and balance tasks. Vibratory stimulation is a potent stimulus for the primary afferents of the muscle spindle. The proprioceptive output can be extracted using vibration to measure the extent of body sway by estimating the center of gravity (CoG) when the vibration is applied. Using this method, it has been reported that the better the proprioceptive function, the greater the sway as a biological response [17]. However, in a previous study, the vibratory stimulation was limited to 60 Hz, which corresponds to the muscle spindle response frequency. Vibratory stimulation at a constant frequency has the disadvantage of not evaluating all organs responsible for proprioception in humans. This is because the organs responsible for proprioception do not depend solely on the muscle spindles; other proprioceptors, such as skin and joint receptors, also play an important role. The afferent response of the muscle spindles is in the range of 20–220 Hz, depending on the muscle condition [18]. A recent clinical study showed that proprioception in response to high frequencies of approximately 250 Hz is significant in the occurrence of lower back pain [19]. Therefore, it is important to evaluate the proprioceptive function using vibratory stimulation at various frequencies [20,21,22]. Hence, we developed a mechanical vibration device that can generate a variety of frequencies (20–300 Hz) that are responsible for almost all proprioceptors evaluating the proprioceptive function of an individual [19]. In this device, local vibratory stimulation at the sweep frequency can be used to exhaustively evaluate the proprioceptors, considering their response frequencies. Publication about guidelines for the use of mechanical vibration stimuli is available [23]. To clinically define a decline in proprioceptive function, it is necessary to develop a reference value based on data obtained from healthy young adults who do not exhibit a decline in proprioceptive function. However, the quantitative evaluation and distribution of the biological responses of healthy individuals to local vibratory stimulation using sweep frequencies remain unclear. Therefore, there are no criteria or cut-off values for determining the decline in proprioceptive function.

Thus, the purpose of this study was to define a new indicator to evaluate the proprioceptive function and propose criteria and cut-off values for estimating functional decline.

## 2. Materials and Methods

### 2.1. Participants

This study was conducted over 12 months (August 2021–July 2022). Written informed consent was obtained from all participants.

A total of 129 healthy young individuals from the Nagoya Heisei College of Nursing and Medical Care, Daido Hospital, Nagoya Institute of Technology, and Nagoya Women’s University were screened. Among them, the measurements of 81 individuals (age: 18–34 years; sex: male (48 years), female (33 years)) were used for the analysis. Missing data (32 individuals who could not be screened according to the protocol, 5 individuals with lower limb or back pain, 2 individuals who staggered during measurement, and 9 individuals who could not detect vibrations and could not perform normal measurements) were excluded.

### 2.2. Design

Inclusion criteria were healthy young individuals aged 18 years or older with no previous orthopedic or neurological disease. Exclusion criteria were orthopedic disease, neuromuscular disease, balance dysfunction, previous surgery for spinal cord disease, and those who could not be measured according to the protocol. Additionally, those who complained of lower leg or lower back pain using the visual analog scale were also excluded.

### 2.3. Device

**Figure 1:**
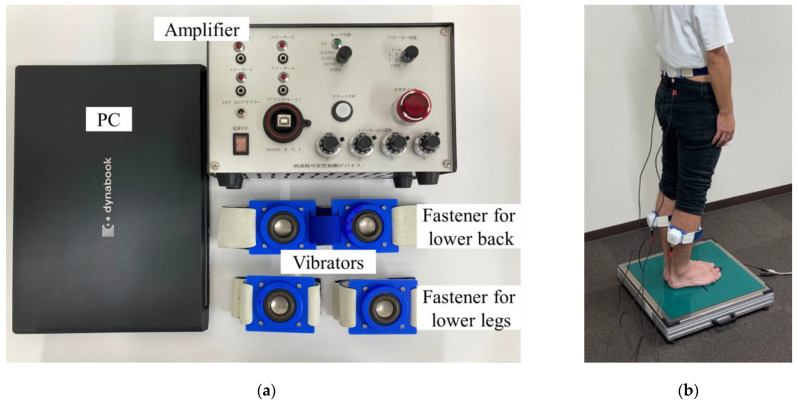
Inspection system for proprioceptive function: (**a**) Variable frequency vibratory stimulation device; and (**b**) experimental setup.

Figure 1a,b show a system for evaluating proprioceptive function. This system consists of a personal computer (PC), an amplifier, four vibrators (NSW1-205-8A, Aurasound, Inc., Santa Fe Springs, CA, USA), three hook and loop fasteners with a holder, and a stabilometer for measuring CoG sway (T. K. K. 5810, Takei Scientific Instruments, Co., Ltd., Niigata, Japan). Using the hook and loop fasteners, one vibrator was attached to each lower leg, and two vibrators were attached to the lower back, as shown in Figure 1b.

The vibration signals were generated using a PC. Mechanical vibrations were then output from the vibrator via an amplifier for mechanical vibratory stimulation. Previous studies have used mechanical local vibration stimulation with amplitudes ranging from 0.4–1.0 mm as proprioceptive inputs [10,17,20]. The vibratory stimulation amplitude was defined as the amount of vertical displacement of the center cap when the speaker was placed horizontally upward. The maximum amplitude that could be output without mechanical vibration distortion at frequencies within 20–300 Hz using the developed device was 0.8 mm. Therefore, a sine wave amplitude of 0.8 mm was selected for the vibration. Additionally, the frequency of the vibratory stimulation could be varied over time in the range of 20–300 Hz. The vibrator was secured to the belly of the gastrocnemius-soleus (GS) and lumbar multifidus (LM) muscles. The gastrocnemius and soleus (GS) muscle groups and the lumbar multifidus (LM) muscles have been reported to play a particularly important role in postural control [7]. The vibrators were mounted on the maximum bulge of the gastrocnemius and soleus for the lower legs and on the lumbar region, on the lumbar multifidus muscle, 4 cm above the superior posterior iliac spine. The contact pressure of the vibrator at the site was adjusted by varying the length of the hook-and-loop fastener. The CoP was measured instead of the CoG. The sampling frequency of this device was 20 Hz, and the time-series data of the CoP coordinates was obtained. The CoP coordinates can be saved as CSV data using the software attached to a stabilometer.

### 2.4. Experimental Procedure

**Figure 2:**
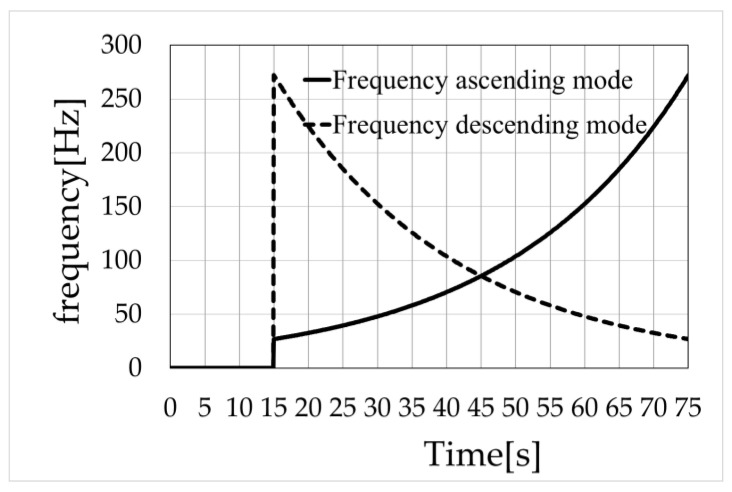
Sweep frequency.

The measurement process was as follows. All participants were asked to confirm that they experienced no pain during the measurements. The CoP was measured on a stabilometer while the participant stood barefoot. The participants were instructed to wear an eye mask and remain still, and measurements were obtained with their feet close together. The participants were instructed to stand with their arms relaxed at each side. To prevent injury from falling during the measurements, one or two researchers stood on either side of the participant and noted whether the participant was falling to provide support in such a case. The sweep frequency developed in a previous study (Figure 2) was used to determine the vibration frequency [19]. The mechanical vibrations were continuously varied from 27 to 272 Hz (frequency ascending mode) or from 272 to 27 Hz (frequency descending mode) for 60 s. The frequency characteristics at a certain time t for the sweep frequency are shown in Equations (1) and (2). The coefficient “a” was set to 0.03851 so that the frequencies at t = 15 and 75 s were 27 and 272 Hz, respectively.

(1) $$f\left(t\right)=\left\{\begin{array}{c} 0\left(0\leq t\leq 15\right)\\ 27e^{a\left(t-15\right)}\left(15\leq t\leq 75\right) \end{array}\right.\;(\mathrm{Frequency}\;\mathrm{ascending}\;\mathrm{mode})$$

(2) $$f\left(t\right)=\left\{\begin{array}{c} 0\left(0\leq t\leq 15\right)\\ 27e^{a\left(75-t\right)}\left(15\leq t\leq 75\right) \end{array}\right.\;(\mathrm{Frequency}\;\mathrm{descending}\;\mathrm{mode})$$

The measurements were taken under two conditions that provided local vibratory stimulation. The gastrocnemius and soleus (GS) muscle groups and the lumbar multifidus (LM) muscles were subjected to vibratory stimulation. Each condition required 75 s, which were divided into two sections. The first 15-second and last 60-second sections were referred to as the pre-section and vibration section, respectively. In the vibration section, the CoP was measured by applying local vibratory stimulation to the GS or LM muscles with the eyes closed. The frequency of the ascending or descending sweep-frequency mode was randomly determined for each individual. A 60-second sit-rest was maintained between GS and LM measurements. During this period, each participant rested on a chair [20].

Next, we describe the proposed analytical method. The proprioceptive function was evaluated using CoP data while applying vibratory stimulation with a sweep frequency. The vibration section was divided into three evaluation sections (ES_i_, i = 1, 2, and 3) that were determined according to the frequency of the local vibratory stimulation and the response frequency of the proprioceptors. Table 1 lists each evaluation section (ES), the corresponding frequency ranges of vibratory stimulation, and the corresponding proprioceptors. The subscript numbers of ES indicate the following correspondence: 1 represents muscle spindles (lower frequency), 30–53 Hz; 2 represents muscle spindles (higher frequency), 56–100 Hz; 3 represents Vater–Pacini corpuscle, 140–250 Hz [21]. As 15 s of data were generally used for CoG sway measurements [1,22], the frequency ranges were determined from Equations (1) and (2) such that the measurement time for each section was 15 s. The reason for this separation of frequency ranges is that the response frequencies of the proprioceptors are different. By defining a more precise cutoff frequency for the decline in proprioceptive function, the frequencies and proprioceptors can be matched to explain which proprioceptors are declining.

Because previous studies have shown a relationship between the anteroposterior displacement of the CoP and the proprioceptive response to vibratory stimulation, only the anteroposterior CoP displacement was considered [22]. Previous studies have used the root mean square (RMS) of the squares of the GS and LM measurements as indicators to evaluate the magnitude of the CoP in the anterior-posterior direction [9,15].

This study used the modified RMS to account for the ability to maintain balance without vibratory stimulation, as shown in Equations (3)–(6). Equations (3)–(5) provide the indicators to evaluate the proprioceptive function for the three frequency ranges ES_1_, ES_2_, and ES_3_, respectively. Because these equations were calculated for each of the two sites with applied vibrations (GS or LM), six indicator values were obtained per individual. Equation (6) indicates the standing-balance ability when no vibration is applied to each of the two sites (GS or LM). In the following equations, the CoP of the vibration section is subtracted from the average value of the CoP of the pre-section for zero-point correction of the CoP of the vibration section based on the CoP of the pre-section.

In the modified RMS, calculating the RMS ratio of the vibration section to the pre-section allowed us to consider the standing balance when no vibration was applied. Evaluating the amount of biological response using the ratio from baseline rather than simply comparing the magnitude of shaking seemed sensible. The greater the sway in each ES in the vibration section compared with that in the pre-section, the greater the value. The better the proprioceptive function, the greater the amount of transition in the anteroposterior direction when shaking is applied [17]. Therefore, a larger value indicated superior proprioceptive function.

(3) $${\mathrm{RMS}}_{*}^{1}=\frac{\sqrt{\frac{1}{N}\sum_{n=n_{1}}^{n_{2}}{\left\{Y_{\mathrm{Vib}\left(*\right)}\left(n\right)-{\bar{Y}}_{\mathrm{pre}\left(*\right)}\right\}}^{2}}}{{\mathrm{RMS}}_{*}^{\mathrm{pre}}}$$

(4) $${\mathrm{RMS}}_{*}^{2}=\frac{\sqrt{\frac{1}{N}\sum_{n=n_{3}}^{n_{4}}{\left\{Y_{\mathrm{Vib}\left(*\right)}\left(n\right)-{\bar{Y}}_{\mathrm{pre}\left(*\right)}\right\}}^{2}}}{{\mathrm{RMS}}_{*}^{\mathrm{pre}}}$$

(5) $${\mathrm{RMS}}_{*}^{3}=\frac{\sqrt{\frac{1}{N}\sum_{n=n_{5}}^{n_{6}}{\left\{Y_{\mathrm{Vib}\left(*\right)}\left(n\right)-{\bar{Y}}_{\mathrm{pre}\left(*\right)}\right\}}^{2}}}{{\mathrm{RMS}}_{*}^{\mathrm{pre}}}$$

where n is the number of data series. $Y_{\mathrm{Vib}\left(*\right)}$ is the CoP in the anteroposterior direction in the vibration section, and ${\bar{Y}}_{\mathrm{pre}\left(*\right)}$ is the average CoP in the anteroposterior direction in the pre-section. The subscript “*” is used to distinguish the location of the stimulator, that is, GS or LM. The subscript number is used to distinguish the ES.

In the equation, N represents the total number of sampled data for each ES, which is equal to 300. This is because all Es were analyzed within 15 s and the sampling frequency was 20 Hz. Table 2 shows the number of data series corresponding to the start and end frequencies of each ES_i_ for both modes of increasing and decreasing frequency. The number of sampling series n corresponding to the measurement time was calculated using Equations (1) and (2), considering the start and end frequencies of each ES. These values were calculated using MATLAB (MathWorks Inc., Natick, MA, USA).

(6) $${\mathrm{RMS}}_{*}^{\mathrm{pre}}=\sqrt{\frac{1}{N}\overset{301}{\underset{n{=}_{1}}{\sum }}{\left\{Y_{\mathrm{pre}\left(*\right)}\left(n\right)-{\bar{Y}}_{\mathrm{pre}\left(*\right)}\right\}}^{2}}$$

The cut-off values representing a decline in proprioceptive function were determined using Equation (7).

Since it has been suggested that individuals with a small sway to vibration have a decline in proprioceptive function [15], a decreased proprioceptive function was estimated when the CoG sway in the anteroposterior direction was below the cut-off values as vibrations were applied.

(7) $${\mathrm{Cutoff}}_{*}^{i}=\bar{{\mathrm{RMS}}_{*}^{i}}-\sigma_{{\mathrm{RMS}}_{*}^{i}}$$

## 3. Results

**Figure 3:**
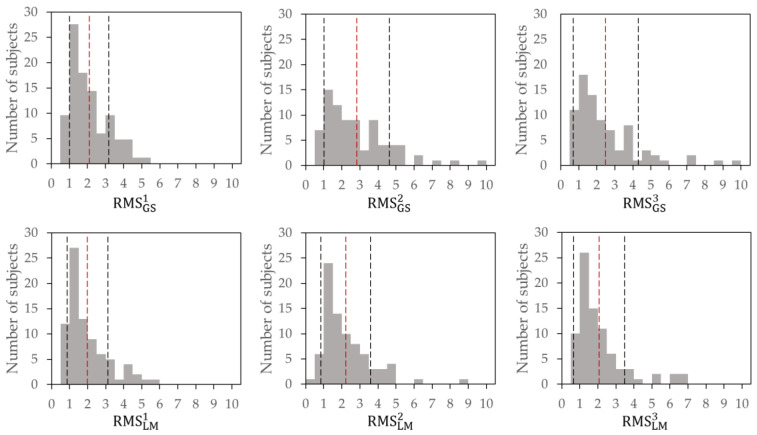
Histogram of ${\mathrm{RMS}}_{*}^{i}$ (*i* = 1, 2, 3, * = GS or LM) for 81 healthy young participants. Abbreviations: RMS, root mean square; GS, gastrocnemius and soleus muscles; LM, lumbar multifidus. ^1^ muscle spindles (lower frequency), 30–53 Hz; ^2^ muscle spindles (higher frequency), 56–100 Hz; ^3^ Vater–Pacini corpuscle, 140–250 Hz.

The individual demographics are presented in Table 3. The histograms of ${\mathrm{RMS}}_{*}^{i}$ (*i* = 1,2,3; * = GS or LM) for the 81 healthy young individuals are shown in Figure 3. The vertical axis indicates the prevalence among the participants. The horizontal-axis interval was set to 0.5. The vertical and horizontal scales of all histograms were aligned. In the graph, the mean value of each indicator is indicated by the red dashed line, and the upper and lower limits of the standard deviation are indicated by the black dashed lines. The left side of the black dashed line represents the cut-off value. Each cut-off value representing a decline in proprioceptive function was defined as the mean minus the standard deviation of each corresponding indicator for proprioceptive function in the participants. 

Table 4 shows the means, standard deviations, and cut-off values for each indicator.

All six indicators exhibited right-skewed distributions. In both the lower legs and lower back, the mean value of ${\mathrm{RMS}}_{*}^{2}$ is the largest, that of ${\mathrm{RMS}}_{*}^{3}$ is the second largest, and that of ${\mathrm{RMS}}_{*}^{1}$ is the smallest. In the same frequency range (ES), the mean value for the lower legs was higher than that for the lower back. For ${\mathrm{RMS}}_{*}^{1}$, the values for all individuals were less than six, but for ${\mathrm{RMS}}_{*}^{2}$ and ${\mathrm{RMS}}_{*}^{3}$, values higher than six were observed for certain individuals. This difference resulted in larger mean values and standard deviations of ${\mathrm{RMS}}_{*}^{2}$ and ${\mathrm{RMS}}_{*}^{3}$.

To analyze the biological response of the participants, the percentages of values below the cutoff are shown in Table 5 for each frequency range and site for the 81 young participants measured in this study.

The percentage was <10% for all frequency ranges and sites. Additionally, the percentage was lower for the lower back (LM) than for the lower legs (GS). Moreover, the percentage of ${\mathrm{RMS}}_{*}^{3}$ functional decline was lower than those of ${\mathrm{RMS}}_{*}^{1}$ and ${\mathrm{RMS}}_{*}^{2}$.

## 4. Discussion

Vibratory stimulation is a potent stimulus for the primary afferents of the muscle spindle. By stimulating endogenous receptors with vibration and evaluating the center of gravity (CoG) affected by the stimulation, proprioceptive functions can be evaluated. Using this method, it has been reported that the better the proprioceptive function, the greater the sway as a biological response [17]. Therefore, it is important to observe the postural response to the application of vibratory stimulation to evaluate the proprioceptive function.

This is the first study that tries to elucidate the distribution of indicators calculated based on the postural response as a biological response when the sweep frequency is applied and provides the criteria and cut-off values for the decline in proprioceptive function. The main finding was that the response to vibratory stimulation of ES_2_ (56–100 Hz) was greater than those of the other ESs when a sweep frequency was applied to the lower legs and lower backs of healthy individuals. ES_1_ (30–53 Hz) and ES_2_ (56–100 Hz) were considered to correspond to the vibratory stimulations of muscle spindles, and ES_3_ (140–250 Hz) to the response frequency of the Vater–Pacini corpuscle. Vibratory stimulation at these frequencies is considered a direct stimulus to the primary afferents of the muscle spindles, causing muscle contraction [18,24]. Previous studies have shown that the activity of muscle spindle primary endings is highest at 80 Hz. The fact that the postural response is higher in the ES_2_ range, which includes the 80 Hz frequency, is in good agreement with previous studies [25]. In the same ES frequency range, the values for the lower legs were higher than those for the lower back. The postural response, as a biological response to vibratory stimulation of the lower legs, may be greater than that to vibratory stimulation of the lower back. The fact that the biological response to the same stimulation differed at different sites indicated that the weighting of the inputs to the proprioceptors was not an exact match [21]. A possible reason for the larger indicator values when vibratory stimulation was applied to the lower legs was the task set. On a stable and hard floor, the ankle strategy, which is more dependent on the proprioceptive signal from the lower legs, can be used against disturbances, whereas on an unstable, soft floor, the hip strategy, which is more dependent on the proprioceptive signal from the trunk, can be used against disturbances [26,27]. In the current study, a postural task was performed on a hard and stable floor that caused a greater response when vibratory stimulation was applied to the lower legs. These results are consistent with those of a previous study that showed a greater postural response to the vibratory stimulation of the lower legs when vibration was applied to the lower legs. In a previous study, in which similar mechanical vibration stimulation was used in young healthy individuals, the amount of response was greater when vibratory stimulation was applied to the lower legs than to the lower back, and this trend was also observed in the present study [28]. In both the lower legs and lower back, the standard deviation of the indicator at ES_3_ was large. This suggests that there are large individual differences in the biological responses when the frequency range of ES_3_, which corresponds to the response frequency range of the Vater–Pacini corpuscle, is applied. In this study, the postural response to the vibratory stimulation of ES_3_ was greater than that of ES_1_. Riemann et al. stated that information from cutaneous and subcutaneous proprioceptors, such as Meissner bodies and Vater–Pacini corpuscles, which act as touch and stretch receptors, respectively, is the sensory source that complements proprioceptor input [29]. This is inconsistent with the results of this study. These discrepancies may have resulted from variations in participant characteristics, such as differences in age groups and body size, as well as differences in the definition and classification of the target criteria and analysis intervals.

Next, we consider the cut-off values shown in Table 4. The cutoff values differed at each site. This indicated that the biological responses induced by each specific receptor were different. The cut-off values were almost equal for ES_1_ and ES_2_ in both the lower legs and back, which could be caused by the differences in the receptors.

The homology between the cut-off values for ES_1_ and ES_2_, but not for ES_3_, may be caused by the differences in proprioceptors. The magnitude of the cut-off value for muscle spindles depends on the site of vibration application and may not depend on the response frequency.

Table 5 shows the percentages of functional decline by site and frequency range for the three frequency ranges. The results show that individuals exhibited a 1.2–9.9% functional decline at each frequency range and site. A percentage decline in proprioceptive function was also observed in healthy individuals. The lower back showed a smaller percentage of decline in proprioceptive function than the lower legs in the areas corresponding to the muscle spindles (ES_1_ and ES_2_). In a previous study, the number of muscle spindles in the lower back was higher than that in the lower legs [30]. A higher number of muscle spindles may have influenced the lower rate of proprioceptive function loss at the sites corresponding to the muscle spindles in the lower back (ES_1_ and ES_2_). The frequency range corresponding to the Vater–Pacini corpuscle (ES_3_) also showed a smaller rate of decline in the proprioceptive function of the lower back. Ito et al. have established that older adults exhibit a declining function of the Vater–Pacini corpuscle in the lower back [31]. Thus, younger adults are less likely to exhibit a decreasing function of the Vater–Pacini corpuscle in the lower back. The present results are similar to those of previous studies in that a few participants had a decline in proprioceptive function in the lower back.

The percentage of functional decline for both the lower legs or the lower back in ES_3_ was smaller than that in ES_1_ and ES_2_. Previous studies [26,27,28] have not reported differences in the susceptibility of muscle spindles or the Vater–Pacini corpuscle to hypofunction in healthy individuals. The results of this study suggest that some healthy participants may have a Vater–Pacini corpuscle with a decline in proprioceptive function. A previous study [31] has suggested that the Vater–Pacini corpuscles in the hips may become hypofunctional with age. This suggests that young, healthy individuals do not have a functional decline in the Vater–Pacini corpuscle of the lower back, and it can be inferred that the Vater–Pacini corpuscle in their lower legs is less likely to be hypofunctional.

This study elucidated the differences in biological responses when vibratory stimulation using a sweep frequency was applied. Moreover, it presented reference and cut-off values that reflect the differences in biological responses for each region and frequency range. The reference and cutoff values may be useful in diagnosing proprioceptive function, and this study could provide information for future research to overcome the common limitations. Although the cut-off values of the indicators of proprioceptive function were validated in this study, it has certain limitations. That is, comparisons by age and data from other regions may be needed to obtain more reliable criteria for determining declining proprioceptive functions. In the future, collecting data on older adults with declining proprioceptive functions would be necessary to validate these cutoff values.

Further validation of the index of proprioceptive function proposed in this study and its cut-off values may enable the identification of declines in proprioceptive function in the future.

## 5. Conclusions

This study defined a new indicator for the quantitative evaluation of proprioceptors using three vibration frequency ranges for the lower legs and lower back, thereby obtaining the characteristics of healthy individuals from the distribution of proprioceptors. The postural response, as a biological response to vibratory stimulation of the lower legs, was greater than that of the lower back. Therefore, the biological response to vibratory stimulation at frequencies corresponding to ES_2_ (56–100 Hz) was greater than that at other frequency ranges. In addition, cut-off values were established as quantitative indexes of proprioceptive function. The percentage of healthy individuals below the cut-off values was found to be higher in the lower legs by site and in the frequency ranges ES_1_ (30–53 Hz) and ES_2_ (56–100 Hz), with a higher percentage of decline in proprioceptive function under the aforementioned conditions. Using the cutoffs in this study, it was possible to determine which of the multiple proprioceptive functions had declined. In both the lower legs and lower back, the rate of decline in proprioceptive function at the sites corresponding to muscle spindles (ES_1_ and ES_2_) was found to be lower in the lower back. The results indicating a low number of individuals with functional decline in the lower back in the present study were consistent with the results of previous studies.

## Figures and Tables

**Table 1:** List of each ES, frequency range of local vibratory stimulation, and corresponding proprioceptors.

$ES_{i}$	Frequency [Hz]	Corresponding Proprioceptors
ES_1_	30–53	Muscle spindles (lower frequency)
ES_2_	56–100	Muscle spindles (higher frequency)
ES_3_	140–250	Vater–Pacini corpuscle

**Table 2:** Number of data series corresponding to the start and end frequencies of ${\mathrm{ES}}_{i}$.

$ES_{i}$	Number of Data Series	Frequency Ascending Mode	Frequency Descending Mode	Corresponding Frequency [Hz]
${\mathrm{ES}}_{1}$	$n_{1}$	350	1450	30
	$n_{2}$	650	1150	53
${\mathrm{ES}}_{2}$	$n_{3}$	680	1120	56
	$n_{4}$	980	820	100
${\mathrm{ES}}_{3}$	$n_{5}$	1156	644	140
	$n_{6}$	1456	344	250

ES: evaluation sections; n: number of data series; The subscript numbers of ES are indicated as follows: 1: Muscle spindles (Lower frequency), 30–53 Hz; 2: muscle spindles (Higher frequency), 56–100 Hz; 3: Vater–Pacini corpuscle, 140–250 Hz.

**Table 3:** Demographic characteristics and functional outcome of the healthy young adults.

Variables	Healthy Young Adults (n=81)
Age, years	22.49 ± 4.13
Height, cm	166.42 ± 8.91
Body Mass, kg	57.77 ± 9.39
BMI, kg/m^2^	20.76 ± 2.18

**Table 4:** Means, standard deviations, and cut-off values of $RMS_{*}^{i}$ with participants.

	$RMS_{GS}^{1}$	$RMS_{GS}^{2}$	$RMS_{GS}^{3}$	$RMS_{LM}^{1}$	$RMS_{LM}^{2}$	$RMS_{LM}^{3}$
Mean	2.09	2.82	2.50	1.99	2.22	2.06
95% CI	1.85–2.33	2.42–3.22	2.10–2.90	1.74–2.24	1.91–2.52	1.75–2.37
Standard deviation	1.08	1.80	1.81	1.14	1.37	1.41
Cut-off value	1.01	1.02	0.69	0.85	0.84	0.65

Abbreviations: RMS, root mean square; GS, gastrocnemius and soleus muscles; LM, lumbar multifidus. ^1^ muscle spindles (lower frequency), 30–53 Hz; ^2^ muscle spindles (higher frequency), 56–100 Hz; ^3^ Vater–Pacini corpuscle, 140–250 Hz.

**Table 5:** Percentage of values below cutoff for each site and frequency range.

	Healthy Young Adults (n=81)
${\mathrm{RMS}}_{\mathrm{GS}}^{1}$	9.9%
${\mathrm{RMS}}_{\mathrm{GS}}^{2}$	8.6%
${\mathrm{RMS}}_{\mathrm{GS}}^{3}$	2.5%
${\mathrm{RMS}}_{\mathrm{LM}}^{1}$	4.9%
${\mathrm{RMS}}_{\mathrm{LM}}^{2}$	4.9%
${\mathrm{RMS}}_{\mathrm{LM}}^{3}$	1.2%

Abbreviations: RMS, root mean square; GS, gastrocnemius and soleus muscles; LM, lumbar multifidus. ^1^ muscle spindles (lower frequency), 30–53 Hz; ^2^ muscle spindles (higher frequency), 56–100 Hz; ^3^ Vater–Pacini corpuscle, 140–250 Hz.

## Data Availability

All relevant data are presented in this manuscript. All the data are available from the authors upon request.
